# Crosstalk of cardiomyocytes and fibroblasts in co-cultures

**DOI:** 10.1098/rsob.150038

**Published:** 2015-06-17

**Authors:** J. Rother, C. Richter, L. Turco, F. Knoch, I. Mey, S. Luther, A. Janshoff, E. Bodenschatz, M. Tarantola

**Affiliations:** 1 Laboratory for Fluid Dynamics, Pattern Formation and Biocomplexity, Am Fassberg 17, Goettingen 37077, Germany; 2 Research Group Biomedical Physics, Max Planck Institute for Dynamics and Self-Organization (MPIDS), Am Fassberg 17, Goettingen 37077, Germany; 3 Institute of Physical Chemistry, University of Goettingen, Tammannstrasse 6, Goettingen 37077, Germany; 4 German Center for Cardiovascular Research (DZHK), Oudenarder Strasse 16, Berlin 13347, Germany; 5 Heart Research Center Goettingen, Robert-Koch-Strasse 40, Goettingen 37099, Germany; 6 Institute of Organic and Biomolecular Chemistry, Georg-August University, Tammannstrasse 6, Goettingen 37077, Germany; 7 Institute of Nonlinear Dynamics, Georg-August University, Friedrich-Hund-Platz 1, Goettingen 37077, Germany

**Keywords:** fibrosis, contractile function, fibroblasts, impedance spectroscopy, electric cell-substrate impedance sensing, cardiomyocytes

## Abstract

Electromechanical function of cardiac muscle depends critically on the crosstalk of myocytes with non-myocytes. Upon cardiac fibrosis, fibroblasts translocate into infarcted necrotic tissue and alter their communication capabilities. In the present *in vitro* study, we determined a multiple parameter space relevant for fibrotic cardiac tissue development comprising the following essential processes: (i) adhesion to substrates with varying elasticity, (ii) dynamics of contractile function, and (iii) electromechanical connectivity. By combining electric cell-substrate impedance sensing (ECIS) with conventional optical microscopy, we could measure the impact of fibroblast–cardiomyocyte ratio on the aforementioned parameters in a non-invasive fashion. Adhesion to electrodes was quantified via spreading rates derived from impedance changes, period analysis allowed us to measure contraction dynamics and modulations of the barrier resistance served as a measure of connectivity. In summary, we claim that: (i) a preferred window for substrate elasticity around 7 kPa for low fibroblast content exists, which is shifted to stiffer substrates with increasing fibroblast fractions. (ii) Beat frequency decreases nonlinearly with increasing fraction of fibroblasts, while (iii) the intercellular resistance increases with a maximal functional connectivity at 75% fibroblasts. For the first time, cardiac cell–cell junction density-dependent connectivity in co-cultures of cardiomyocytes and fibroblasts was quantified using ECIS.

## 1. Background

Cardiovascular ischaemic heart diseases are the leading causes of natural death worldwide. The occurrence of this disease is tightly linked to the heterocellular electromechanical crosstalk between myocytes and non-myocytes of the heart. Furthermore, cardiac extracellular matrix (ECM) guides popularization and scarring dynamics of infarcted heart tissue. As the associated clinical picture of fibrosis represents a serious problem in medical treatment, garnering a deeper understanding of the interplay between fibroblasts (Fb), cardiomyocytes (CM) and their substrates is pivotal to improve therapeutic methods and thus living conditions for patients with myocardial infarction.

It is well known that CM have limited regeneration capabilities [1] and make up more than two-third of the heart by volume [2], but only about one-third by number, while regenerative non-myocytes, such as Fb and endothelial cells, outnumber CM by a factor of two [3] with much less volume. Assuming the regenerative function of Fb after cardiac infarction, the development of cardiac fibrosis as disease depends on the keloid translocation of Fb into infarcted necrotic tissue [4]. There, an elevated production of ECM components, especially collagen, a remodelling of cell-matrix contacts and further reorganization of the ECM occur, accompanied by differentiation of Fb into cardiac myofibroblasts [5]. This gradual differentiation process has been observed in *in vitro* co-cultures from primary tissue and leads to decreased contractile behaviour of CM [6].

In the context of this work, we refer to *coupling* as the contractile behaviour displayed in the oscillatory impedance time series reflecting cell-shape changes over time, while *connectivity* represents the barrier resistance of a cell monolayer arising from cell–cell contact formation and expression, and therefore reflects intercellular communication through gap junctions and adherens junctions. An additional major consequence of the transformation into a myofibroblast is the modulated connectivity between CM and Fb. During fibrosis, remodelling occurs of homo- and heterocellular cell–cell contacts, which were formed by desmosomes, cadherins and connexins, especially Cx43 [6–10]. In previous studies on co-cultures of Fb and CM, both electrical and mechanical communication aspects have been studied by monitoring conduction velocity (CV), action potential duration [11], re-entrant activity [12], spiral wave dynamics [13], gap-junctional diffusion and gene activity [14], striation level and force generation [15] or transmission [16], and electromechanical feedback [17]. In the latter study, modulated tension between myocytes and myofibroblasts resulted in activation of mechanosensitive channels, which in turn impaired conduction. In this study, we systematically quantify the crosstalk between Fb and CM, investigating three major aspects relevant for co-cultures with variant Fb content: (i) adhesion to substrates as a function of elasticity and surface chemistry, (ii) dynamics of contractile motion comprising mechanical coupling, and (iii) electromechanical connectivity.

The Fb–CM co-cultures are usually studied by means of optical microscopy [18,19] or dynamical gap-FRAP experiments employing labelled connexins [11]. These techniques have the inherent disadvantages of being time consuming and invasive due to the need for extensive labelling. Therefore, we propose a different approach here based on non-invasive electric cell-substrate impedance sensing (ECIS) that is capable of monitoring minuscule cell-shape and cell–substrate distance changes with nanometre sensitivity and variations of the barrier resistance between cells in real time. ECIS was first developed by Giaever & Keese [20–23] and is often employed for studying adhesion, spreading or proliferation of cells [24–26]. It can be used to analyse single cells or confluent monolayers cultured on gold electrodes integrated in culture wells and provides continuous spectral information of the complex electrical resistance *Z* (or impedance) as cellular dynamics restrict the flow of weak electrical currents. However, so far only individual research groups have applied ECIS for the analysis of co-cultures: these studies include model systems of cell invasion, wound healing [27–29], extravasation [30] or the blood–brain barrier [31]. More recently, noise originating from adherent cells has been used to assess dynamic properties of cellular ensembles that give rise to collective morphological fluctuations [32]. So-called micromotion of cells measured by resistance fluctuations was successfully used to quantify cell vitality [33,34] as well as metastatic cellular potential [35,36]. In the first ECIS studies on primary rat CM and stem-cell derived CM, ECIS was employed as a tool to screen cytoplasmic resistivity effects of TNF-α or compounds modulating beating frequency [37–41].

Here, collective phenomena of periodic contraction waves of Fb–CM co-cultures are inferred from impedance oscillations through coupling analysis. Additionally, the barrier function of adherent confluent co-cultures is monitored by recording frequency-dependent impedance data subsequently modelled by the transfer function of an electrical equivalent circuit [22,42]. We observe that the beat frequency decreases nonlinearly with increasing fraction of Fb, while the intercellular resistance increases. Thereby, we are able to provide a comprehensive electromechanical picture of adhesion kinetics, beating coupling and connectivity in Fb–CM co-cultures.

## 2. Material and methods

### 2.1. Cell culture preparation

**Figure 1.**
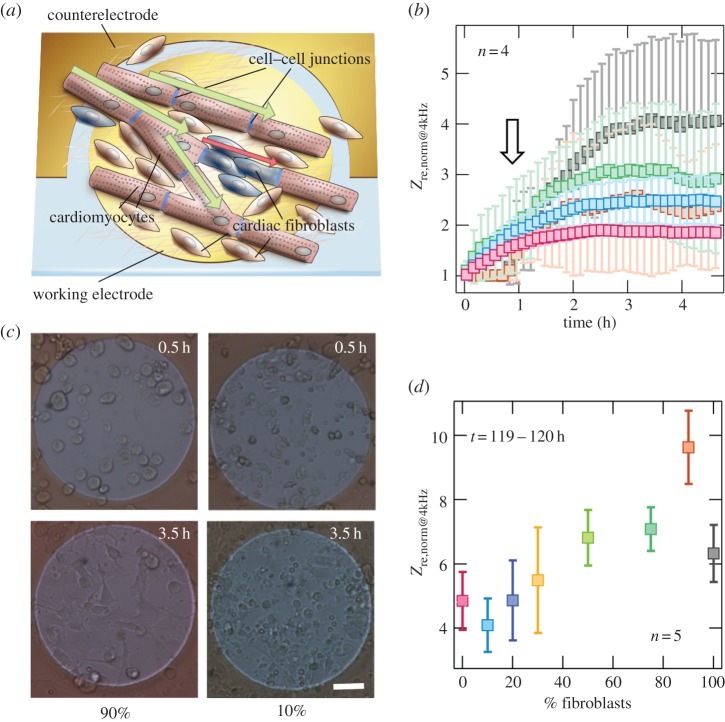
Adhesion and growth: *Z*_re,norm@4kHz_ time courses. (*a*) Scheme of experimental set-up: Fb–CM co-cultures are seeded onto functionalized circular gold electrodes geometrically restricted by an insulator layer. Cells serve as insulators to the electrical current and *Z*_re,norm@4kHz_ represents their insulating capability. Electromechanical connectivity arises through anchoring via gap- and adherens junctions. (*b*) Mean time series of impedance showing initial adhesion after seeding of Fb–CM co-cultures: 0% (pink), 10% (light blue), 75% (dark green), 90% (orange) and 100% (grey) Fb at 200 000 cells per well inoculum. Impedance is normalized to its initial value before cell addition. Given for *n* = 4 ± s.d. The arrow points to the initial time shift in impedance increase between low and high Fb ratios. (*c*) Representative bright field micrographs for 90 and 10% Fb in Fb–CM co-culture taken 0.5 h and 3.5 h after addition of cells with triangular cells (Fb) and cells appearing oval to longitudinal (CM); scale bar is 40 µm. (*d*) Mean *Z*_re,norm@4kHz_ values for Fb fractions of 0, 10, 20, 30, 50, 75, 90 and 100% after 5 days of growth for the given interval, where a stationary impedance level is reached (electronic supplementary material, figure S1*b*), given for *n* = 5 ± s.d.

The method for CM isolation was described elsewhere [19]. Briefly, for our experiments, we used solutions of neonatal ventricular CM and cardiac Fb, as well as consequential mixtures of specific cell seeding concentrations. Therefore, we prepared the hearts of 1–2-day-old neonatal Wistar rats. The isolated, minced ventricles underwent several enzymatic digestion steps with collagenase II (300 U ml^−1^, Worthington, USA), each for 15 min at 37°C. Tissue preparations for cardiac Fb were digested with 0.05% trypsin/EDTA for 20 min at 37°C in combination with glass pearls and under constant stirring. After counting, the cells were diluted appropriately in Dulbecco's modified Eagle medium/F12 (DMEM/F12, Gibco, Germany) containing 10% bovine serum and 1% penicillin/streptomycin. Cell suspensions were seeded directly onto functionalized ECIS chambers until a final volume of 500 µl containing 25 to 500 × 10^5^ cells per well (see electronic supplementary material, figure S1*a*) was reached. For short-time adhesion and spreading studies (measurement less than 8 h, figure 1*a*,*b*; electronic supplementary material, figures S1*a* and S2, as well as table S1), the two primary cell types were plated simultaneously. In long-time assays (more than 100 h incubation), multilayer formation was largely prevented by adding Fb 24 h after CM inoculation.

### 2.2. Gelatine coating

For the comparison of different electrode functionalizations (see electronic supplementary material, table S1 and figure S2), ECIS chambers were pre-coated with four elastic substrates consisting of gelatine solutions covering ratios of 0.002% up to 2% and fibronectin solutions with ratios of 0.1 to 5% (v/v%) (both Sigma-Aldrich, Germany). Pre-coating solutions were incubated for at least 1–2 h at 37°C. For ECIS recordings, the electrode arrays were placed in an incubator and the cells were cultured on the electrodes under physiological conditions (37°C, 5% CO_2_, humidified atmosphere) for up to 5 days (see electronic supplementary material, figure S1*b*) to ensure an optimum of connectivity and a two-dimensional monolayer. Medium exchange was performed daily and from day 3 onwards was switched to DMEM/F12 containing 6% bovine serum, 1% penicillin/streptomycin and 1% norepinephrine.

### 2.3. Electric cell-substrate impedance sensing, inter-beat interval (IBI) and beating frequency

Adhesion, spreading and monolayer formation of Fb–CM co-cultures on gold electrodes were monitored by recording time-resolved impedance at 4 kHz excitation frequency using an ECIS ZΘ set-up equipped with 8W1E electrode array chips that have circular electrodes of 250 µm diameter (both Applied Biophysics, Troy, NY, USA). *Z*_re,norm@4kHz_ was normalized to the impedance level of cell-free electrodes and the frequency of 4 kHz chosen due to the best S/N ratio. In addition, we adopted capacitive monitoring of electrode occupancy at high frequencies (64 kHz) yielding separate spreading rates -*S*_1_ and -*S*_2_ for CM and Fb in the co-culture (see electronic supplementary material, figure S2) [26].

Besides impedance time series recorded at a fixed frequency, we simultaneously measured changes of the barrier function of the adherent co-culture layer by determining changes in the barrier resistance *R*_b_, the membrane capacitance *C*_m_ and the impedance contribution from the cell-substrate cleft *α* (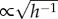 with *h* the distance between cell and the substrate). Frequency-resolved impedance readings (10–10^5^ Hz) were subjected to equivalent circuit fitting based on an area contact model (see figure 3*a*). Following Giaever *et al.* [22,42,43], *Z*_re,norm@4kHz_ was normalized to the impedance level for electrodes immersed in buffer, while the parameter *R*_b_, quantifying the degree of connectivity via adherens and gap junctions, was normalized to the electrode area. As the transfer function relies on fully established cell monolayers, time traces were analysed 40 h after seeding of the cells (see electronic supplementary material, figures S3 and S4). For details, refer to electronic supplementary material.

To analyse contractile motion and therefore beating coupling of heart cells in Fb–CM co-cultures, real part of the complex impedance was collected for at least 5 min at an AC-frequency of 4 kHz with a sampling rate of 30 Hz. The impedance data were low-pass filtered using a moving average filter (window size = 5 samples). Afterwards, the intervals between subsequent peaks in the detrended |*Z*| signals were calculated using a peak detection algorithm (peakdet.m from http://www.billauer.co.il/peakdet.html (21 March 2015)) in Matlab (MATLAB and Statistics Toolbox 2012b, The MathWorks, Inc., Natick, MA, USA). Then, beating frequencies were calculated as the inverse of the interval time. All Fb–CM fractions were measured in triplicate. In addition, exemplary power spectral densities were calculated using fast Fourier transformation from impedance time series after linear detrending (see electronic supplementary material, figure S1*c*).

### 2.4. Atomic force microscopy (AFM)–Hertz indentation analysis of electrode coating

Mechanical properties of the substrates immersed in ECIS medium were analysed by force-mapping indentation experiments employing a MFP3D (Asylum Research, Santa Barbara, CA, USA) with Veeco Cantilevers (MLCT, C-Lever, nominal spring constant *k* = 0.01 N m^−1^). Data evaluation (fitting of the contact model) in Matlab followed a procedure outlined by Schaffer *et al.* [44] employing the Sneddon model assuming a conical indenter shape.

The substrates were immersed in the same medium also used for ECIS measurements. To evaluate the homogeneity of the substrates, force mapping of an area of 20 × 20 µm^2^ was carried out with one force indentation curve per micrometre step. In total, at least three force maps were collected per substrate at different positions.

### 2.5. Optical microscopy and image analysis

Immunostaining, fluorescence microscopy, bright field phase-contrast microscopy, propagation-induced phase-contrast (PIPC) imaging and phase mapping were applied to visualize electrode coverage as well as characteristic morphological and dynamical features of each cell type’s cytoskeleton. Therefore, co-cultures were grown on the ECIS culture dishes or comparable culture dishes for 5 days until a tight two-dimensional layer was formed. After washing twice with PBS and carrying out permeabilization in 0.1% Triton-X-10 solution for 20 min, *α*-actinin, *α*-SMA, vimentin, N-cadherin (N-Cad) and connexin-43 (Cx43) and DAPI staining was performed at room temperature as detailed in the electronic supplementary material.

An upright fluorescence microscope (Olympus BX51 with 20, 40 or 100× water immersion objectives with NA = 0.2, 0.8 or 1.0, respectively, Germany), equipped with a colour CCD camera (Olympus DP71) was used for visualization of the staining directly onto the gold electrodes after removal of the culture wells (electronic supplementary material, figure S5). High-resolution characterization of fluorescence immunolabelling was done with a confocal laser-scanning microscope (Olympus FluoView1000, 60× oil immersion objective) with imaging array (see figure 4).

Based on PIPC imaging as described previously [45], it was possible to visualize the cell activity by means of contraction. For the phase-contrast, we used a 5 W LED as partial coherent light source and a 0.3-mm pinhole for reduction of numerical aperture. Through a pair of condensing lenses, the field of view is brought to a macroscopic scale. Due to the fact that the refraction changes during contraction, it is possible to visualize wave propagation. In the analyses, a filter in space and time, as well as a normalization filter were applied.

Phase singularity (PS) statistics are well established for analysing excitable media [46]. Phase singularities are centres of wave emission and can be identified by the spatial analysis of the phase of the periodic signal. PS statistics are considered as local positions inside phase, where the actual activation state of the medium is not defined. Therefore, singularities are assumed to be the origin of excitation waves and indicator of wave break-ups or spiral cores. In order to detect and analyse singularities, data signals have to be converted into phase data, which is done by Hilbert transformation based on time series. By calculating the path integral over the gradient of phase around a singularity, the topological charge of the detected singularities was defined, representing also the direction of rotating activity. Through the use of a nearest neighbour method for adjacent frames, it was possible to track singularity paths and thus to show the movement of wave break-ups and spiral cores. To avoid false positive detection, only singularities with a lifetime of more than five frames were taken into account.

The optical connectivity assays relies on a self-written analysis tool for CLSM images based on Python (www.python.org) and is composed of two parts: the first determines the pixel amount of each colour in an overlayed and crosstalk corrected, but otherwise not modified image containing all labelled channels, and normalizes to the total amount of pixels, based on individual channel threshold filtering. The second part determines boundaries: our aim here is to know what fraction of yellow pixels borders areas of red, green or both pixels (e.g. cell types). We therefore count the amount of red and green pixels in the vicinity (*N* nearest neighbour pixels) of each yellow boundary pixel. The algorithm computes the sum of green and red pixels adjacent to yellow borders and compares it to a specific colour noise threshold parameter. Only if the sum exceeds this parameter will it be counted as corresponding to cell interface.

## 3. Results

### 3.1. Adhesion, spreading and growth of fibroblast–cardiomyocyte co-cultures

Spreading, adhesion and growth of cells can be monitored non-invasively and time dependently by impedance spectroscopy. The method allows measuring the distance of cells to the electrode (cell–substrate distance) as well as the connectivity between cells (cell–cell contacts) employing ultrasmall electrodes immersed in regular culture media. Depending on the frequency, impedance is either dominated by cell–substrate contact or cell–cell contacts. Therefore, the nonlinear increase in impedance due to cell-specific properties of CM and Fb permits analysis of cell mixtures with regard to spreading and adhesion kinetics (figure 1*a*). Mean time courses of the normalized real part of impedance at 4 kHz excitation frequency *Z*_re,norm@4kHz_ for the first 4.5 h after seeding various co-culture mixtures are presented in figure 1*b* and the electronic supplementary material, figure S1*d*. In general, we found that with increasing Fb ratios, the real part of the complex impedance increases indicating a stronger insulating capability of Fb as opposed to CM.

Representative micrographs for 90 and 10% Fb in a Fb–CM co-culture taken 0.5 and 3.5 h after addition of cells reveal the electrode coverage underlying the monitored impedance increase (figure 1*c*). While CM were found to adhere and spread slowly after 30 min, the majority of Fbs adhere later but once attached spread out faster (see 3.5 h). The delay in increase of impedance within the first hours of culture correlates well with a high fraction of Fb in the mixture, as highlighted by the arrow in figure 1*b*. Adhesion to functionalized electrodes with various elastic gelatine mixtures and densities of fibronectin was tested for growth optimization as also done previously [26] (summarized in electronic supplementary material, table S1 and figure S2).

A quasi-stationary state of impedance indicating formation of an electrically tight two-dimensional layer was reached when cells were cultured on the electrodes for more than 4 days (electronic supplementary material, figure S1*b*). Figure 1*d* summarizes mean *Z*_re,norm@4kHz_ values for all measured Fb fractions reached within an interval of 119–120 h after seeding: there is a clear trend towards higher *Z*_re,norm@4kHz_ levels for increasing fractions of Fb in the co-culture, with a maximum at 90% Fb.

### 3.2. Coupling determination in fibroblast–cardiomyocyte co-cultures: electric cell–substrate impedance sensing and propagation-induced phase-contrast imaging-based contractile motion analysis

**Figure 2.**
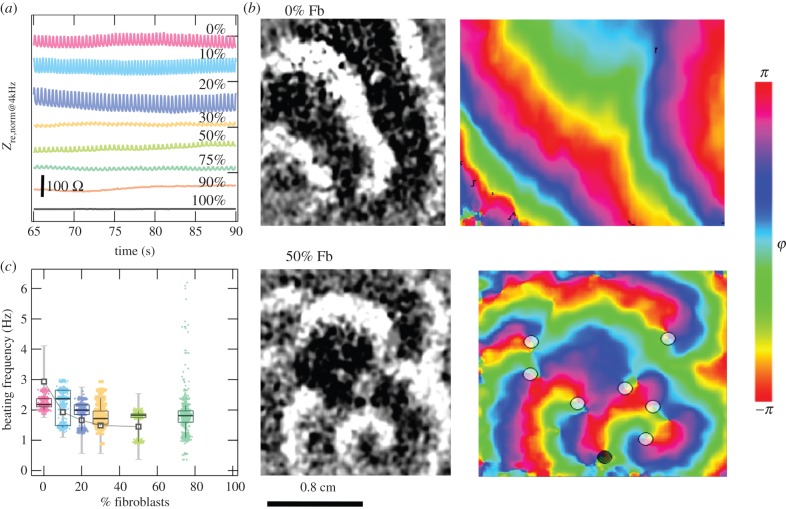
Beating coupling: beating frequency and PIPC analysis. (*a*) Exemplary time-dependent impedance signal of the resistance for different Fb fractions (0% (pink), 10% (light blue), 20% (purple), 30% (yellow), 50% (light green), 75% (dark green), 90% (orange), 100% (grey)), (*b*) PIPC imaging, exemplarily shown for 0 and 50% Fb ratios, and phase space analysis for both corresponding mixtures shown for phase *φ* shifts from *π* to −*π*. (*c*) Analysis of beating frequency of Fb fractions from ECIS assay (box plots with median value, 25 and 75% percentiles and 10 and 90% whiskers) or, as shown in (*b*), assessed via PIPC (grey data points and lines ± s.d.) Both assays given for *n* = 3. Samples with Fb fractions more than 75% did not show any beating behaviour.

In the context of this work, we refer to *coupling* as the contractile behaviour displayed in the oscillatory impedance time series reflecting cell-shape changes over time. The coupling of co-cultures was evaluated by measuring the beating frequency and fidelity displayed in impedance data. Figure 2*a* shows high-resolved time traces of *Z*_re,norm@4kHz_ for all measured Fb fractions 4 days after seeding. To analyse beating properties, we determined the temporal interval between two succeeding beats over time. From these, the beating frequency was determined as shown in figure 2*c*. Beating of CM influenced current flow, which resulted in either an increase or a decrease of *Z*_re,norm@4kHz_ independent of the Fb fraction. Oscillations were attributed to a closer or more remote distance of the basal membrane from the electrode. Samples containing high CM portions exhibited higher oscillation amplitudes of the impedance signal, which we attributed to a larger number of beating CM cells on the electrode. As we used 250 µm diameter electrodes, the number of cells per electrode in mean corresponded to 32 cells.

In addition to the ECIS approach (which lacks spatial resolution of local activation patterns), we also applied optical mapping with respect to visualizing the distribution of local activation patterns with higher spatial resolution. Our experiments were done on a macroscopic imaging set-up based on the technique of PIPC [47], which allows the optical mapping of excitation wave propagation on a scale of several centimetres (see figure 2*b* for two exemplary Fb ratios). Due to the fact that refraction changes during cell contraction, it is now possible to illustrate wave dynamics by taking difference images. Besides, PIPC image information was also filtered in space and time by use of Gaussian moving average filter. In order to show the influence of different fibroblast concentrations on the wave propagation, we computed time series over one pixel within the observed region of the confluent cell monolayer (figure 2*c*). Afterwards, the images were analysed with regard to PS statistics, which was done by applying a Hilbert transform filter and computational recognition of zero crossings in phase [46]. Doing this, we found an increase of PS with rising fibroblast percentages and thus indications for a gradual shift from quasi-planar wavefronts (0% Fb) to more complex vortex-like patterns (50% Fb).

ECIS and PIPC contractile motion results are summarized in figure 2*c*. Both methods show the same trend and clearly confirm that beating frequency decreases with an increasing fraction of Fb in the co-culture. As the PIPC approach captures break up of a planar wavefront into spiral waves, we strongly believe that the information collected with ECIS indeed also captures population-based activity patterns. Only ECIS was sensitive enough to detect contractile motion of the 75% Fb mixtures.

It should be mentioned here that although mapping the contraction dynamics shows, owing to the calcium contraction coupling, a good correspondence to intracellular calcium dynamics, it does not allow conclusions concerning electrical excitation signals (i.e. membrane voltage) or effects on coupling among all three signals. Therefore, simultaneous measurements of membrane voltage, intracellular calcium and contraction could give information about disturbances in the excitation–contraction coupling. Nevertheless, the visualization of changes in spatio-temporal contraction dynamics is a perfect method to detect rough influences of co-cultural effects in a non-invasive way.

### 3.3. Connectivity in fibroblast–cardiomyocyte co-cultures: electric cell–substrate impedance sensing model

**Figure 3.**
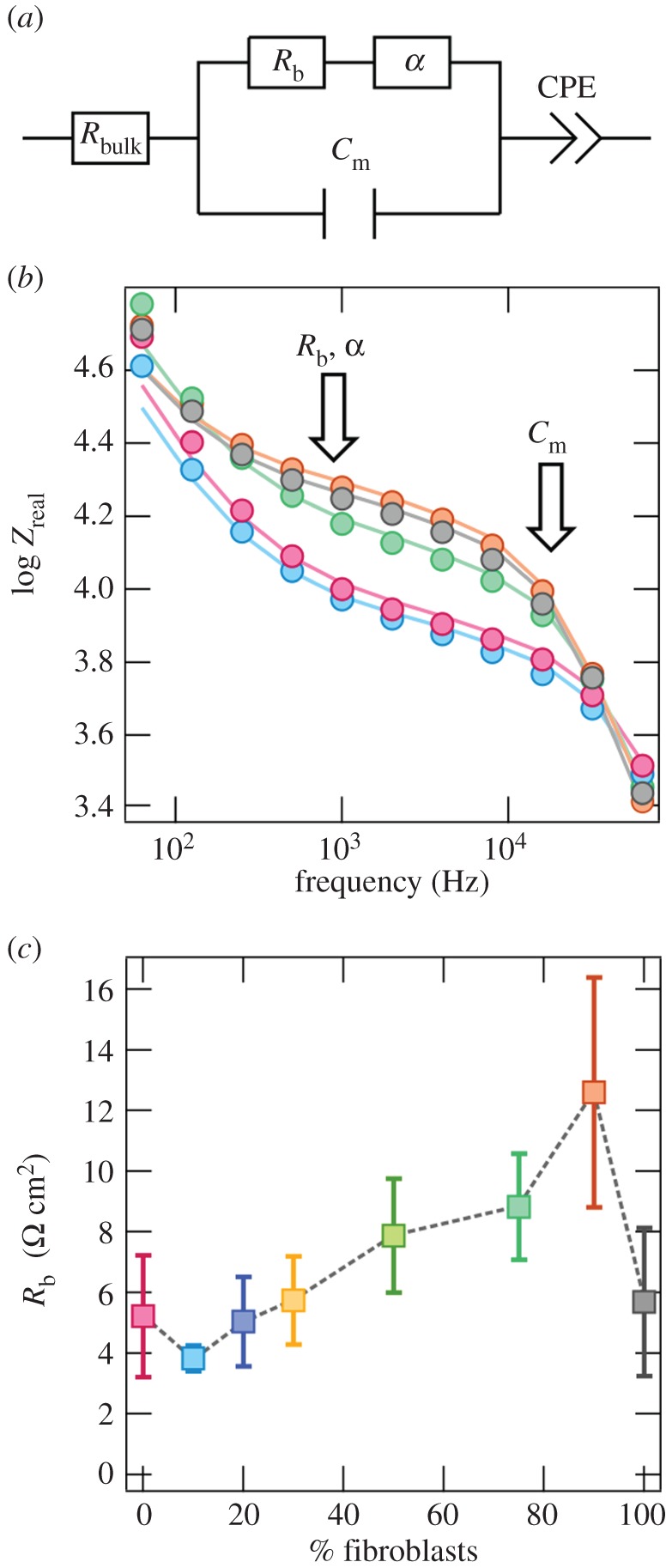
Connectivity: ECIS model. (*a*) Equivalent circuit model applied to experimental impedance spectra with *R*_bulk_ (resistance of culture medium), *R*_b_ (barrier resistance between cells), *α* (specific electrolyte resistance in the subcellular cleft), CPE (constant phase element, *viz.* impedance at the electrode–medium interface) and *C*_m_ (membrane capacity). (*b*) Frequency regimes important for each cellular parameter described under (*a*) (highlighted by arrows) in a logarithmic real part impedance spectra *Z*_Re_ (dots) and corresponding nonlinear regression (lines) recorded 120 h after seeding of Fb–CM with 0% (pink), 10% (light blue), 75% (dark green), 90% (orange) and 100% (grey) Fb (electronic supplementary material, figure S3*a*: imaginary impedance spectra). (*c*) Mean values for the cell–cell contact density parameter *R*_b_ over quasi-stationary 119–120 h interval, given for *n* = 5 ± s.d. Connectivity quantified via *R*_b_ increases with increasing Fb fractions (for *C*_m_ and *α*: electronic supplementary material, figure S3*c*,*d*).

In the context of this work, we refer to *connectivity* in terms of the barrier resistance of a cell monolayer arising from cell–cell contact formation and expression. Connectivity analysis was realized through a modified contact area ECIS model that treats the cells as two-dimensional dielectric objects (adopted from Lo & Ferrier [42], figure 3*a*). In this study, cells were assumed to be rectangular with circular caps at the shorter side. The following width to length relation was assumed based on optical inspection of 50 different cells: CM 64 × 22 µm^2^, Fb 72 × 22 µm^2^; therefore, a mean length of 68 µm was used for both cell types. The following reasonable parameters describing electrical components of the set-up and the medium were adopted from the literature [42]: specific electrolyte resistance (*ρ* = 54 Ω × cm), specific capacitance of the bare electrode (*C*_n_ = 12 µF cm^−2^) and a capacitive constant phase element (CPE = 200 (μF/cm)^−*n*^) with an exponent of *n* = 0.95. Three parameters were fitted to the impedance spectra (figure 3*b* and electronic supplementary material, figure S3*a*) obtained from the co-culture mixtures: (i) the barrier resistance *R*_b_, representing cell–cell contacts; this resistance can be attributed to the intercellular cleft and is, in the case of Fb–CM co-culture mixtures, determined by adherence junctions, desmosomes and gap junctions; (ii) *C*_m_, the membrane capacitance; and (iii) *α*, the specific electrolyte resistance of the subcellular cleft, which is proportional to 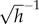, with *h* being the cell–substrate distance.

Time series of *R*_b_, *C*_m_ and *α* are presented in the electronic supplementary material, figure S4. Note that the Lo model is solely valid for completely covered electrodes (represented by *C*_m_) and for cells expressing cell–cell contacts (represented by *R*_b_). Therefore, time traces started at least 40 h after seeding, where microscopically observable confluence was reached. For 119–120 h, strongest connectivity and therefore highest *R*_b_ 12.6 Ω × cm^2^ was found for 90% Fb–CM mixtures, which was a significantly higher barrier resistance than for other co-cultures with *R*_b_ < 6 Ω × cm^2^ (figure 3*c*). Note that this suggests that even though *R*_b_ is maximal at 90% Fb, only mixtures up to 75% Fb with an *R*_b_ of 8.6 Ω × cm^2^ still sustain collective beating as found by IBI coupling analysis. The connectivity of a co-culture consisting of 90% Fb is furthermore higher by a factor of 2–3 than the connectivity of monocultures of CM (5.2 Ω × cm^2^) or of neat Fb (5.7 Ω × cm^2^), thus pointing to a cooperative effect (refer also to electronic supplementary material, figure S3*b*).

Concomitantly, we also found a decrease of membrane capacitance *C*_m_ from a value of 1.05 µF cm^−2^ for pure CM to 0.85 µF cm^−2^ for 90% Fb in the mixture. A decrease in total membrane area usually gives rise to this smaller capacitance, which can be explained by a smoother membrane surface of Fb or ongoing transformation processes ([36]; electronic supplementary material, figure S3*c*). Additionally, only for the case of 90% Fb–CM, we found a significant increase in *α* by 1 Ω^1/2^ × cm as compared with monocultures, corresponding to a decrease in cell–substrate distance or conductivity in the cleft between the Fb membrane and the electrode (electronic supplementary material, figure S3*d*).

### 3.4. Connectivity in fibroblast–cardiomyocyte co-cultures: immunohistochemistry

To relate the gained connectivity quantification to the presence of cells on the electrodes and the occurrence of cell–cell contacts, composition of co-culture mixtures was imaged via immunohistochemistry. Exemplarily, epifluorescence microscopy images of CM in Fb–CM mixtures for all fractions used are shown in the electronic supplementary material, figure S5 (staining for sarcomeric *α*-actinin and DAPI). Images were recorded directly onto ultrasmall ECIS gold electrodes with a top-view water immersion objective for each applied mixture and verify a regular phenotype and the expected distribution.

**Figure 4.**
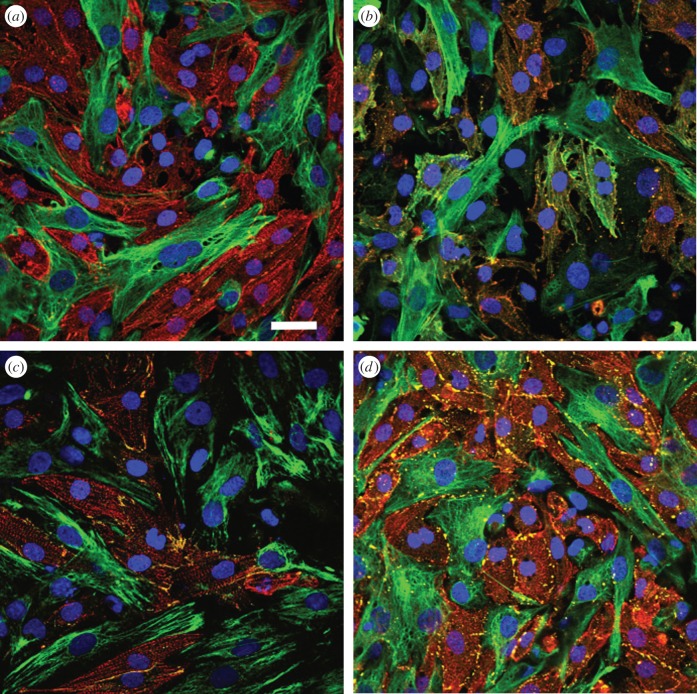
Connectivity: immunohistochemistry. (*a*) Confocal fluorescence micrographs of the co-culture with threefold staining of *α*-actinin in CM (red), vimentin in Fb (green) and nuclei via DAPI (blue). (*b*) Confocal micrographs with threefold staining of *α*-actinin (red), *α*-SMA in Fb (green) and nuclei (blue). (*c*,*d*) Confocal micrographs of *α*-actinin (red), vimentin (green), nuclei (blue) and either of two major cell–cell adhesion molecules (yellow): adhesion junction N-Cad or gap-junctional Cx43. In (*a*–*d*), co-distribution is exemplarily shown for 80% Fb; scale bar is 30 µm.

Confocal fluorescence micrographs of co-cultured two-dimensional monolayers are given in figure 4*a*,*b*. They show the distribution of each cell line within the confluent monolayer using *α*-actinin and vimentin (A) or *α*-SMA and vimentin (B); *α*-actinin relates again to CM, *α*-SMA indicates the presence of non-myocytes, while vimentin is a Fb marker; thus, the heterogeneous distribution of Fb points to a distribution as found for diffusive fibrosis *in vivo*. The samples in figure 4*c*,*d* exemplarily show staining of the adhesion junction molecule N-Cad and the major gap-junction protein Cx43 after co-staining with DAPI, *α*-actinin and vimentin. In these fluorescence micrographs, both cell–cell junction molecules could clearly be identified between CM–CM pairs, but also between Fb–Fb pairs as well as between Fb–CM couples. Micrographs thereby support the presence of all three possible combinations of cell junctions. We have also assessed both the absolute occurrence of N-Cad and Cx43 with *increasing* Fb portions (not shown) as well as their relative occurrence between cell pairs (electronic supplementary material, figure S6); we do not find a significant change in the absolute number of N-Cad or Cx43 pixels with increasing Fb ratios (fluctuating around 0.2–2% with a mean of 0.9%), but we do observe an increase of the relative occurrence of both cell junction molecules in Fb–Fb pairs (relative to total pixel amount), which corresponds to the increase in *R*_b_ quantified via connectivity analysis, thus pointing to a higher level of electromechanical connectivity.

## 4. Discussion

In the present impedimetric study, we investigated the impact of the Fb fraction in Fb–CM co-cultures on (i) adhesion to modified substrates, (ii) contractile coupling, e.g. beating amplitude and frequency, and (iii) electromechanical connectivity.

We found optimal adhesion for Fb and CM on gelatine substrates with an elasticity of 7 kPa at low Fb content mixtures, which is in good agreement with the 5–18 kPa previously observed for CM monocultures [15,48,49] on PAA gels. At high Fb content mixtures, preferential adhesion of Fb was shifted to stiffer substrates with Young’`s moduli of 11.5 and 48.4 kPa, which is in good agreement with the preference of Fb–CM co-cultures (2 : 1 ratio) for 50 kPa substrates [16]. In addition, we showed that the density of fibronectin of 0.1 to 0.5% results in highest spreading rates of CM, especially for mixtures with high Fb fractions. Although we found strong striation levels for all substrate modifications, our non-optical assay allowed a clear differentiation of the cell–substrate interaction in a very early stage of adhesion, within the first 4.5 h of seeding. Shi *et al.* [47] measured elasticity of cells cultured on various substrates using AFM indentation experiments. They found cell elasticity of 12–13.5 kPa (CM) and 5–15 kPa (Fb), which is of the same substrate elasticity regime that we report to be optimal for adhesion in this study. Therefore, the presented data support the common notion in the literature that cell adhesion occurs with highest efficiency on substrates with comparable stiffness to that of the cells [15,48]. Spreading rates extracted from initial impedance readings correspond, by the same magnitude, to those found in the literature for adhesion of epithelial cells to laminin or vimentin [26]. Additionally, we observed a time lag between adhesion of CM and adhesion of Fb of 0.5–3.5 h optically and non-optically. Therefore, we believe that the spreading rates computed in the first linear regime represent mainly the adhesion/spreading behaviour of CM of *Z*_im,norm@64kHz_, while especially the spreading of Fb prevails in the second linear regime.

Taking a closer look at the beating frequency results, we find a strong agreement with previously published work on beating behaviour of CM and Fb–CM co-cultures *in vitro*: Hu *et al.* [46] observed an increase in beating frequency for primary rat CM monocultures upto 48 h of incubation at a frequency above 2 Hz; for Fb–CM, in general, with increasing number of Fb, a decrease in frequency of beating is found. Vasquez *et al.* [11] compared infarcted and non-infarcted Fb in co-cultures and described via optical techniques, whole cell patch clamp, Cx43 immunochemistry and gap-FRAP kinetics a decrease in beating behaviour accompanying an increase in connexin expression (factor of 2.5). This study also describes a decrease in beating coupling, but furthermore we were able to quantify *C*_m_, *α* and intercellular connectivity via *R*_b_. *C*_m_ was found to be of the order of 1.05 µF cm^−2^ for pure CM, a value also found previously elsewhere [41]. An *α* of 2.5 Ω^1/2^ × cm was comparably described in the literature for pure CM and was related to absolute cell–substrate distances of 59 nm [38,50]. Concerning *R*_b_, we found an increase by a factor of 2.5–3 (12.6 Ω × cm^2^) as compared with the monoculture of Fb (5.7 Ω × cm^2^). The *R*_b_ value for the monoculture of Fb is a factor of 19 higher compared with normal Fb (0.3 Ω × cm^2^) [2,42,51], thereby hinting at a transformation process. Thompson *et al.* [17] found reduced electric conduction due to mechanical coupling. In a combined computational and experimental work, Zlochiver *et al.* focused on re-entry spiral wave dynamics: there, for the first time five to eight different Fb–CM fractions have been analysed optically via phase maps. With increasing Fb fractions, rotational frequencies and phase singularities as well as CV were found to be reduced, but therefore higher Fb coupling coefficients needed to be assumed [12]. We could experimentally show both an increase in cellular connectivity and an exponential frequency decrease concomitant with increasing Fb fractions. Furthermore, the presence and relative occurrence of N-Cad- and Cx43-mediated cell–cell connections confirms an electrical and mechanical nature of the quantified connectivity.

Finally, Miragoli *et al.* [52] described an activation threshold of CM to Fb spatial distributions of more than 15.7% in their study on ectopic activity in co-cultures. The beating coupling assay in this study shows that a mixture with a ratio of Fb to CM above 3 : 1 (75% Fb) is unable to retain contractile behaviour, while maximal connectivity can be found for 90% Fb, implying a threshold for beating activation in between these two mixtures.

In essence, our study clearly shows that the decrease in beating frequency goes along with an increase in electromechanical connectivity, and we are able to quantify this finding systematically and for the first time non-optically employing non-invasive impedance spectroscopy.

## 5. Conclusion

Combining results from adhesion, coupling and connectivity analysis, we can claim the following. (i) We have for the first time employed impedimetric spreading rates to detect differential cell spreading in co-cultures. This procedure was extended to also account for the influence of substrate elasticity. We confirmed a preferred adhesion regime for substrate elasticity around 7 kPa for low Fb content mixtures of Fb–CM co-cultures, which we found to be shifted up to 48 kPa substrates with increasing Fb fractions. (ii) By employing for the first time eight different co-culture ratios, the electromechanical beating coupling, e.g. contractile frequency, was found to decrease in a nonlinear fashion with increasing Fb fractions in the mixture as confirmed by two independent assays. (iii) Following the ECIS model, intercellular resistance *R*_b_ was found to increase with increasing Fb fractions. Results also hint at a cooperative electromechanical connectivity, as the Fb fractions of 75 and 90% display *R*_b_ elevated by a factor of 2–3 compared with the connectivity in monocultures of CM or Fb. Note that the mixture with the highest Fb fraction where we are still able to detect oscillations via period analysis is 75%. This is the same mixture that holds the second highest connectivity.

Therefore, we are able to detect and also quantify non-optically a gradual decrease in beating frequency accompanied by an increase in connectivity with increasing number of Fb in various Fb–CM mixtures.

## Supplementary Material

Electronic Supporting Material
