# Supplementary material for: Crosstalk of cardiomyocytes and fibroblasts in co-cultures
Source: Open Biol. 2015 Jun 17;5(6):150038. doi: 10.1098/rsob.150038 (PMC4632504; doi:10.1098/rsob.150038)
Supplement: Electronic Supporting Material [file rsob150038supp1.docx]

**Electronic Supporting Material**

**ESM Text**

**Electric Cell-substrate Impedance Sensing (ECIS)-Contact area model.**

Frequency-resolved impedance readings (10-10^5^ Hz) were subjected to equivalent circuit fitting derived from an area contact model (see Fig. 3A). Following Giaever *et al.* ([1-3](#_ENREF_1)), *Z*_re,norm@4kHz_ was normalized to the impedance level of cell-free electrodes while the parameter *R*_b_, quantifying the degree of connectivity via adherens and gap junctions, was normalized to the electrode area. As the algorithm relies on formed cell-cell contacts, time traces were only analyzed starting 40 h with respect to seeding time (see supp. Fig. 3 and supp. Fig. 4). In detail, we refer to the following set of four existing formulas to fit the real and imaginary contributions to the total complex impedance *Z*_c_ of the equivalent circuit according to *Z*_c_= Z_real_ + *Z*_imag_ :

(1) $R_{b}^{rec}=\frac{\frac{4L}{W}+2\pi}{\frac{4L}{W}+\pi}R_{b}$

(2) $\gamma=\left(\frac{2\alpha}{W}\right)\sqrt{\frac{1}{Z_{n}}+\frac{1}{Z_{m}}}$

(3) $Z_{m}=\frac{1}{(i\omega C_{m})}$

(4)


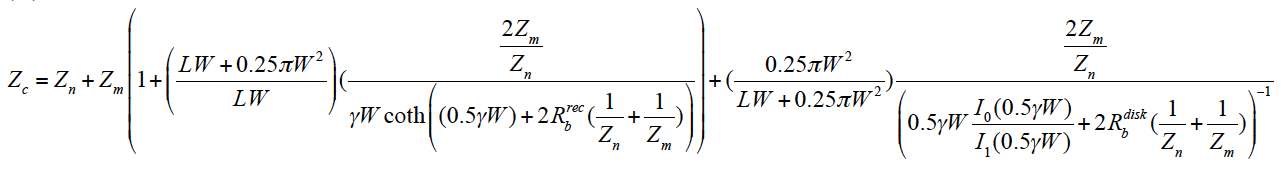


with *W* = Cell width, *L* = Cell length, *R*_b_^rec^ = Transcellular resistance for rectangular cell geometry, *R*_cons_= (*ρ* × 0.25*A*)/*r*, *r* = electrode radius, *A* = electrode area, *ρ* = specific electrolyte resistance, *C*_n_ = bare electrode capacitance, *C*_m_ = cell membrane capacitance, *Z*_m_ = Impedance of cell covered electrode, *Z*_n_ = Impedance of the bare electrode and *I*_0_ and *I*_1_ are two bessel functions of first order.

**Optical microscopy-Immunostaining.**

The cells were washed two times with PBS, nonspecific binding sites saturated with 5 % FCS/0.2 % Tween-20 in PBS for 30 min, washed with PBS twice again, and then incubated with primary antibody solutions according to the manufacturers recommendation. Monoclonal mouse anti-*α*-actinin IgG1 (Sigma-Aldrich, Seelze) was added for 60 min, then washed twice with PBS, followed by a second incubation period of 60 min either with anti-vimentin, anti-*α* –SMA, anti N-cadherine or anti-connexin-43 monoclonal mouse IgG1 antibodies (Sigma-Aldrich, Seelze).

Subsequently, two PBS washing steps were included followed by a 45 min. treatment with secondary antibodies. We used polyclonal DyLight-488-conjugated goat-anti mouse IgG1 antibodies (Vector Laboratories Inc., Burlingsome, CA, USA) for staining of *α*-actinin (CM-marker), *α*-SMA (Fb marker) or the intermediate filament vimentin (Fb-marker), while N-Cadherine (adherens junctions) and connexin-43 of gap junctions (marker for cell connections) were visualized with DyLight-549-conjugated anti mouse IgG1 antibodies (Dianova, Jackson Immuno research Lab, PA, USA); for multiple stainings, sometimes *α*-actinin was stained with DyLight-648-conjugated goat-anti mouse IgG1 antibodies. Finally, after washing twice with PBS, labeling of the DNA/nuclei visualization was carried out by staining for 20 min with 4,6 diamidino-2-phenyindole (DAPI, Sigma-Aldrich, Seelze).

**Adhesion: Substrate elasticity and anchoring sites**

Adhesion to functionalized electrodes with various elastic gelatine mixtures and densities of fibronectin were tested. Table S1 summarizes the results in terms of spreading rates for 10% and 75% mixtures (%Fb in Fb-CM co-culture), as determined from initial linear regime of the imaginary (or capacitive) impedance at 64 kHz frequency *Z*_im,norm@64kHz_ ([4](#_ENREF_4)): this frequency was chosen since the imaginary part of the impedance is inversely proportional to the electrode coverage (supp. Fig. 2). Two different spreading rates could be determined within the first 4.5h of culturing. These we attributed to individual spreading phase of each cell line in the co-culture. According to images shown in Fig. 1C, the first linear phase could be attributed to adhesion of CM (-*S*_1_ ≥ 0.2h^-1^), the second to Fb (-*S*_2_ ≤ 0.19h^-1^). Best CM adhesion, thus lowest spreading rates of -*S*_1_ = 0.38 ± 0.005h^-1^ (low Fb content) or -*S*_1_ = 0.85 ± 0.048h^-1^ (high Fb content) were observed for gelatine mixtures of 0.02%. They showed a young modulus of 7.2 kPa as determined by AFM indentation experiments using the Hertzian contact model. Gelatine mixtures of 2% hamper initial CM adhesion, as can be inferred by the two -*S*_1_ values of 0.14 and 0.15h^-1^. Fb adhered preferentially to hard surfaces in high content mixtures with Young’s moduli of 11.5 kPa and 48.4 kPa or -*S*_2_ rates of 0.19 and 0.18h^-1^. At low Fb contents, soft substrates with young moduli of 1.6 kPa and 7.2 kPa were preferred. Three of four spreading rates for both cell types and mixtures were minimal for 0.02% gelatine. Also three out of four minima were found for 0.5% fibronectin functionalization, which probably bears the optimal combination of elasticity and adhesion sites. Note that modification of the fibronectin content did not affect detectable Young`s modulus of the gelatine gel.

**ESM Figures and captions**


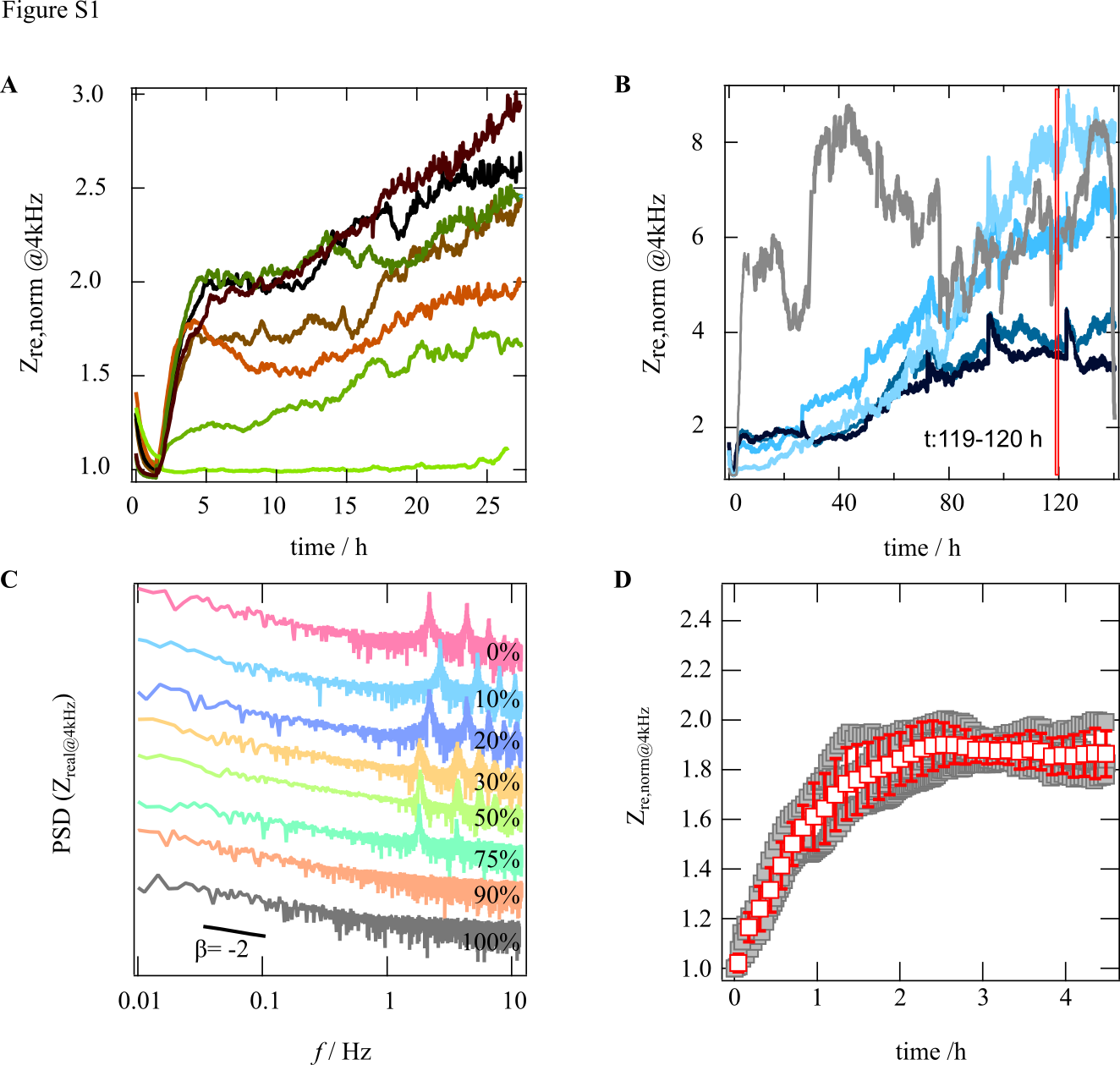


**Figure S1.** *CM monoculture and Fb-CM co-cultures:* **A** *Z*_re,norm@4kHz_ time courses showing cell density dependent increase due to adhesion, spreading and monolayer formation of CM-monocultures. Inoculi from 25 to 500 ×10^5^ cells are shown (25 (■), 100 (■), 150 (■), 200 (■), 300 (■), 400(■), 500 (■)×10^5^  in 0.5 ml per well). For the first 30 h of incubation, a maximum is found for 200.000 cells. **B** Full *Z*_re,norm@4kHz_ time courses shown for 120 hours of incubation with an inoculum of 200.000 cells/well (■ 0% ■ 20% ■ 75% ■ 90% ■ 100% Fb in Fb-CM). Light microscopic monolayer formation is already observed within first 24 h, remodeling of cell shape/size and connectivity accounts for continuous impedance increase up to 100 hours. **C** Power spectral densities (PSD) of the time traces shown in Fig. 2A. **D** Extraction of mean time courses of the normalized real part of impedance at 4kHz excitation frequency *Z*_re,norm@4kHz_ for the first 5 hours after seeding exemplary shown for 100% CM after interpolation routine, given for *n* = 4 ± S.D.


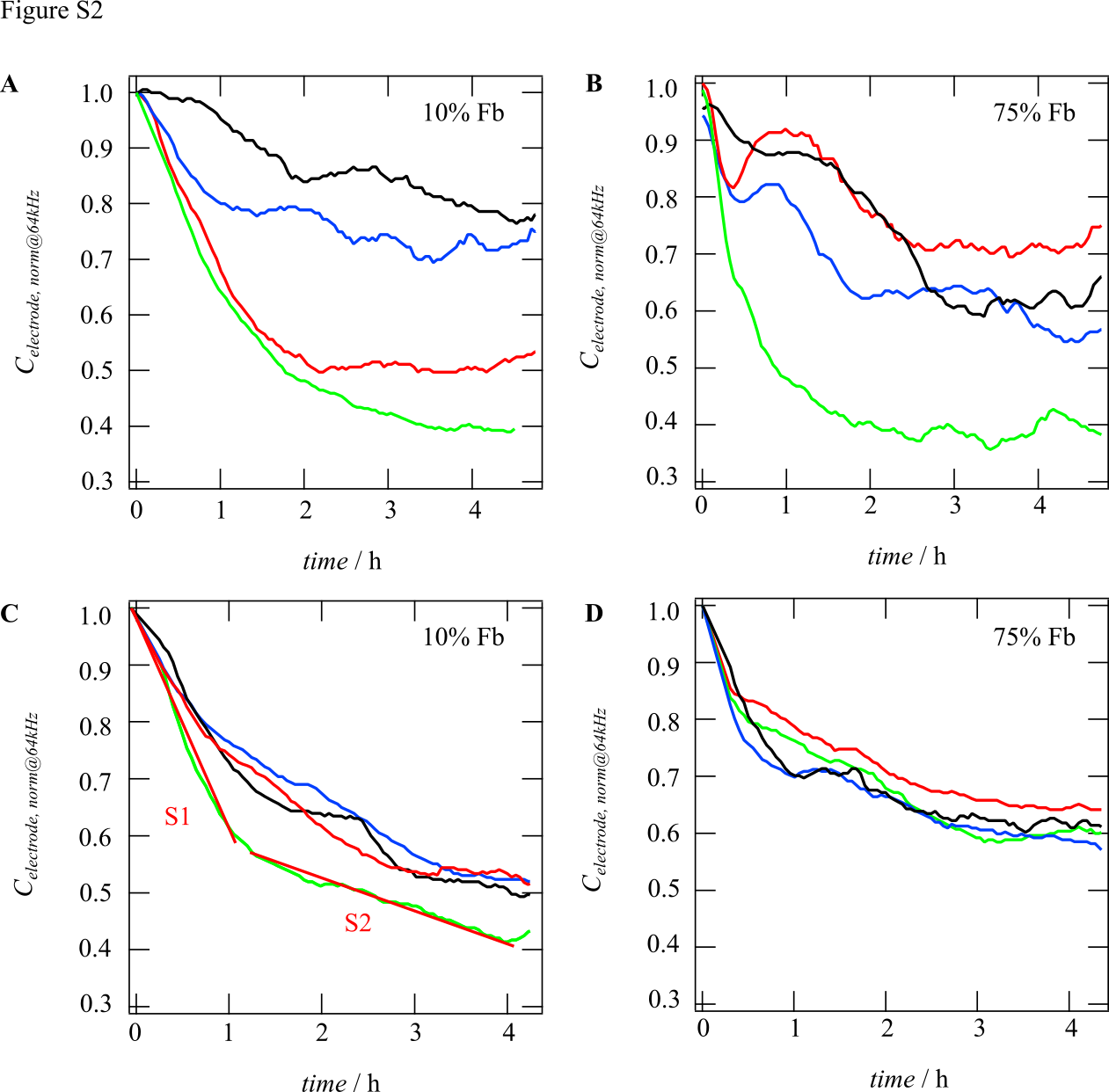


**Figure S2.** *Adhesion: Substrate elasticity and anchoring sites:* Initial adhesion time courses of *Z*_im,norm@64kHz_ showing two distinct regimes (red lines in C) fitted linearly to gain spreading rates -*S*_i_. **A** Functionalization with various gelatine mixtures at low Fb density (10%): ■ 2%, ■ 0.2%, ■ 0.02%, ■ 0.002%. **B** Functionalization with various gelatine mixtures at high Fbt density (75%): ■ 2%, ■ 0.2%, ■ 0.02%, ■ 0.002%. **C** Functionalization with 0.02% gelatine and various fibronectin densities at low Fb density (10%): ■ 5%, ■ 1%, ■ 0.5%, ■ 0.1%. **D** Functionalization with 0.02% gelatine and various fibronectin densities at high Fb density (75%): ■ 5%, ■ 1%, ■ 0.5%, ■ 0.1%.


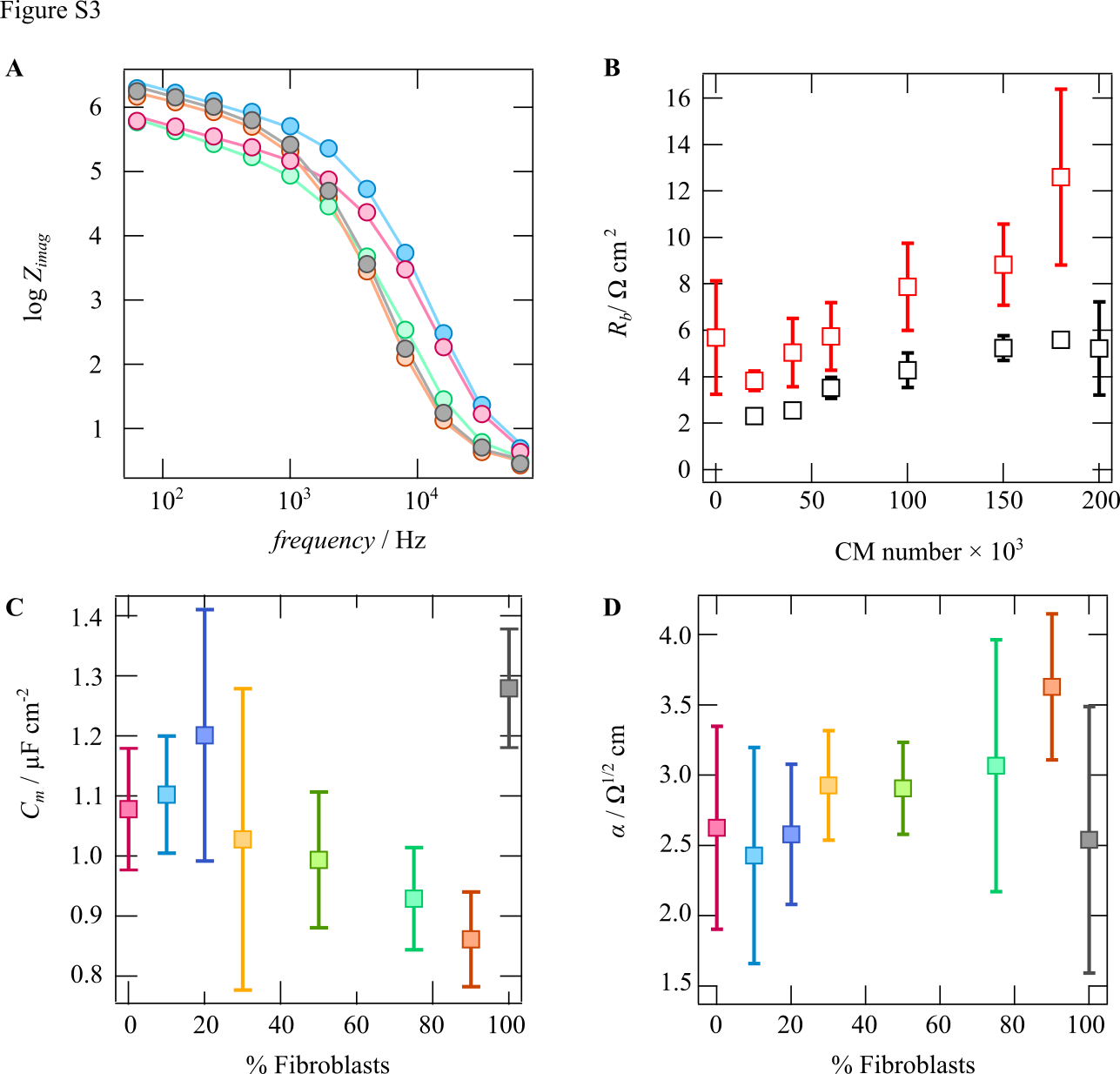


**Figure S3**. *ECIS model fit,* *CM monocultures and Fb-CM co-cultures*: **A** ECIS model imaginary fit results for various mixtures corresponding to Figure 3 B. **B** Connectivity analysis of CM monocultures (■) with comparison to the connectivity in co-cultures (■), *i.e.* 20, 40, 60, 100, 150 and 180 ×10^3^ cells. Mean values for *C*_m_ (**C**) and *α* (**D**) over the quasi-stationary 119-120 h interval, given for *n* = 5 and ± S.D., colour code corresponding to Fig. 3C. While connectivity quantified via *R*_b_ increases (refer to Fig. 3 C), membrane capacitance *C*_m_ decreases with increasing Fb fraction and *α* shows no significant mixture-dependent trend except for an increase for 90% Fb.


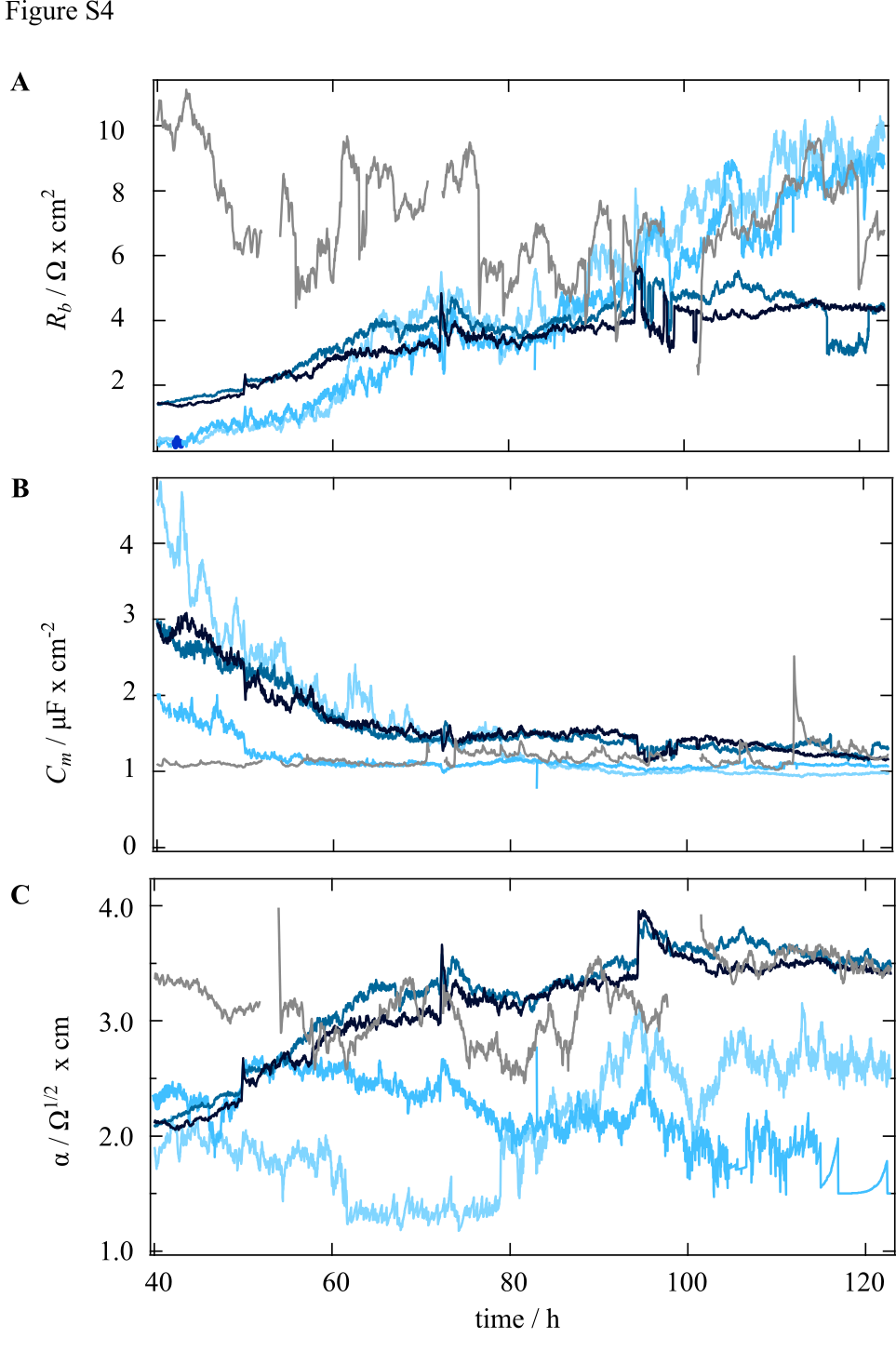


**Figure S4**. *ECIS-model: Time courses of R*_b_ (**A**)*, C*_m_ (**B**), *α* (**C**). Time series for the fitting parameters *R*_b_, *C*_m_ and *α* for the same exemplary co-culture as under supp. Fig. 1B (refer to color code there).


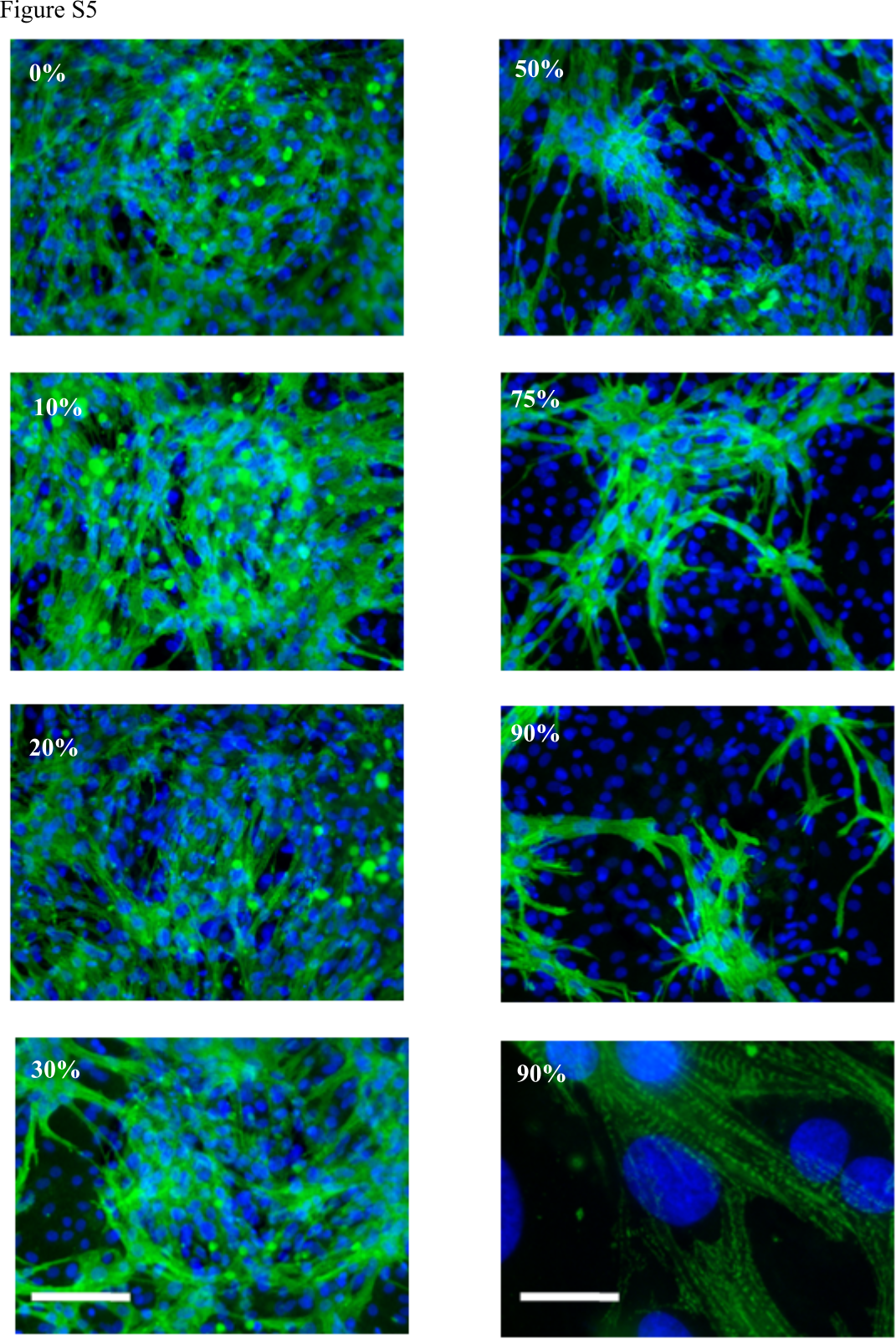


**Figure S5**. *Immunohistochemistry-Distribution of CM and Fb on ECIS-Electrodes*: Fluorescence light microscopic images displaying distribution of CM in all co-culture monolayers used after staining of α-actinin via secondary antibody conjugated to DyLight-488 and nuclei of CM and Fb via DAPI. Holes in the 0% category can be attributed to the 2% non-CM or Fb cells as a result of *ex-vivo* isolation, *viz.* mainly endothelial cells. Micrographs were recorded 120 h after cell seeding on electrodes with functionalization of 0.02% gelatine and 5% fibronectin. Magnification for 90% highlights striation level found in all CM regardless of the fractions of Fb used. Scale bar corresponds to 100 µm in all images besides the magnification, where it is 20 µm.

**Figure S6**. *Immunohistochemistry-Distribution of N-Cadherine and Connexin 43*: Localization of cell-cell adhesion molecules in relation to the total pixel amount between neighboring pairs of cells: Both N-Cad and Cx43 are located between Fb, CM and in Fb-CM pairs. With increasing Fb ratios, both the N-Cad and Cx43 mediated the-Fb coupling increases (to 80% of all neighboring cell-cell contacts for N-Cad image series, to 60 % for the connexin 43 series), *n* = 17 cells per category.

**ESM-Table**

**
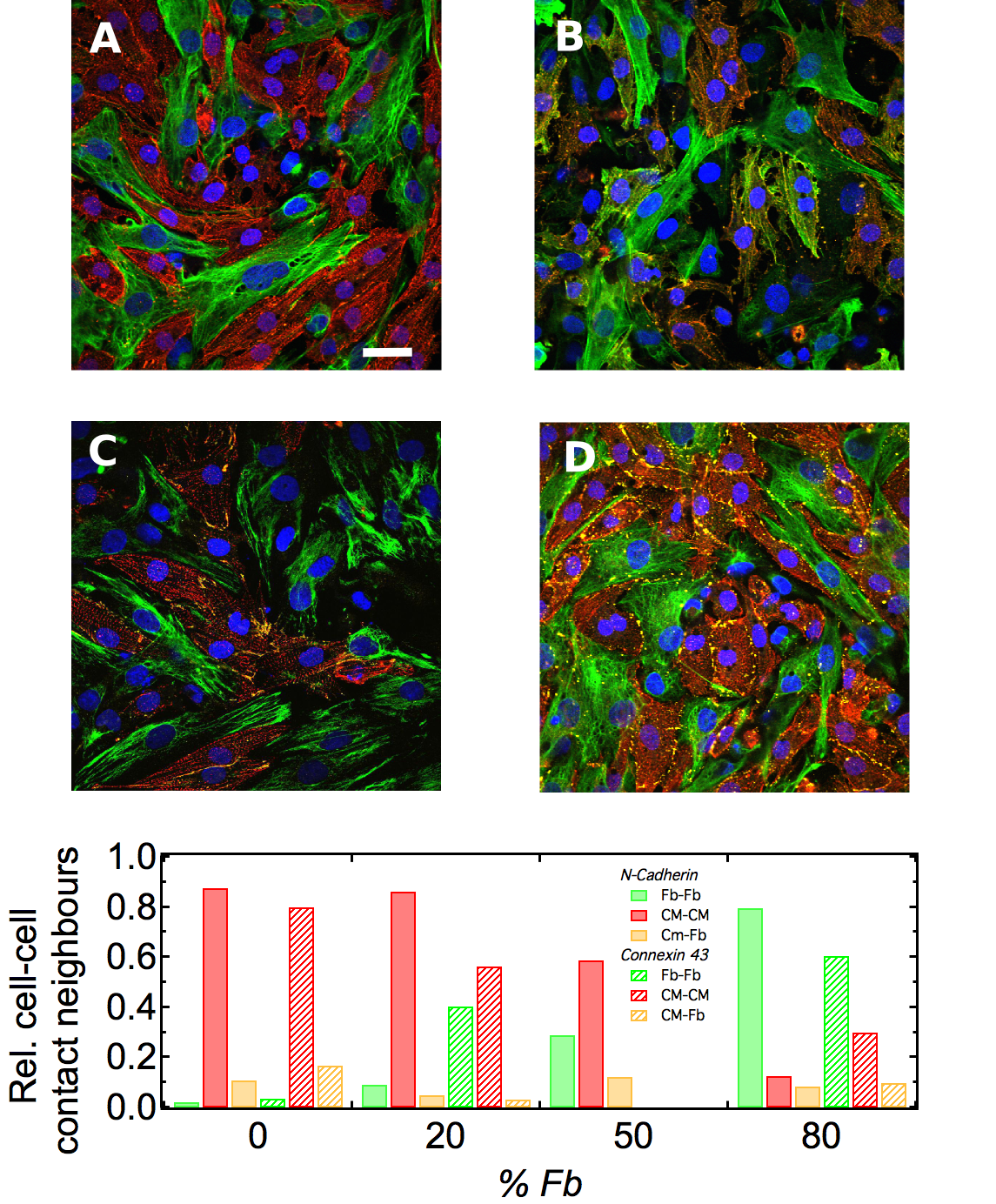
Table S1.** *Adhesion: Substrate elasticity and anchoring sites.* Spreading rates -*S*_i_ ± S.D.(h^-1^) determined at 64 kHz from the two initial linear regimes of *Z*_im,norm@64kHz_ after functionalization of electrodes with gelatine and fibronectin of various compositions. E-modulus ± S.D. in kPa for thin gelatine layers extracted from Hertz model after AFM indentation.

| **Substrate** | | **10% Fb** | | **75% Fb** | |
| --- | --- | --- | --- | --- | --- |
| *%Gelatine* | *E-module* / kPa | -*S*_1_ ± SD / h^-1^ | -*S*_2_± SD / h^-1^ | -*S*_1_± SD / h^-1^ | -*S*_2_± SD / h^-1^ |
| 0.002 | 1.6 ± 0.4 | 0.34 ± 0.008 | 0.16 ± 0.010 | 0.58 ± 0.053 | 0.12 ± 0.004 |
| 0.02 | 7.2 ± 1.5 | 0.38 ± 0.005 | 0.17 ± 0.003 | 0.85 ± 0.048 | 0.09 ± 0.005 |
| 0.2 | 11.5 ± 4.2 | 0.22 ± 0.007 | 0.05 ± 0.003 | 0.31 ± 0.024 | 0.19 ± 0.005 |
| 2 | 48.4 ± 24 | 0.14 ± 0.004 | 0.07 ± 0.002 | 0.15 ± 0.011 | 0.18 ± 0.005 |
| *%Fibronectin* | *E-module* / kPa | -*S*_1_ ± SD / h^-1^ | -S_2_± SD / h^-1^ | -*S*_1_± SD / h^-1^ | -*S*_2_± SD / h^-1^ |
| 0.1 | - | 0.25 ± 0.007 | 0.12 ± 0.002 | 0.38 ± 0.044 | 0.07 ± 0.002 |
| 0.5 | - | 0.35 ± 0.015 | 0.05 ± 0.001 | 0.39 ± 0.048 | 0.09 ± 0.001 |
| 1 | - | 0.21 ± 0.009 | 0.09 ± 0.001 | 0.21 ± 0.040 | 0.07 ± 0.001 |
| 5 | 7.2 ± 1.5 | 0.20 ± 0.009 | 0.15 ± 0.010 | 0.28 ± 0.014 | 0.06 ± 0.005 |

**ESM-Literature**

1. Giaever, I., Keese C. R. 1991 Micromotion of Mammalian-Cells Measured Electrically. *Proc Natl Acad Sci USA* **88**(17):7896-900.

2. Lo, C. M., Ferrier J. 1998 Impedance analysis of fibroblastic cell layers measured by electric cell-substrate impedance sensing. *Phys Rev E* **57**(6):6982-7.

3. Lo, C. M., Keese C. R., Giaever I. 1995 Impedance analysis of MDCK cells measured by electric cell-substrate impedance sensing. *Biophys J* **69**(6):2800-7.

4. Wegener, J., Keese C. R., Giaever I. 2000 Electric cell-substrate impedance sensing (ECIS) as a noninvasive means to monitor the kinetics of cell spreading to artificial surfaces. *Exp Cell Res* **259**(1):158-66.
